# Awareness of the Connection Between Food and Nutrition in Adolescents: A Pilot Study

**DOI:** 10.3390/nu17121949

**Published:** 2025-06-06

**Authors:** Shihkuan Hsu, Shih-Yao Liu

**Affiliations:** 1 Center for Teacher Education, National Taiwan University, Taipei City 106319, Taiwan; 2 Department of Pediatrics, National Taiwan University Hospital, Taipei City 100225, Taiwan; syliu017322@ntu.edu.tw

**Keywords:** food and nutrition knowledge, association of nutrition and lifestyle, awareness, secondary school students, adolescents

## Abstract

Background/Objectives: Adolescence is an important period for developing the knowledge and skills for healthy eating. Past studies have found that declarative knowledge of food and nutrition alone is not sufficient to improve healthy behavior, but how adolescents make connections from factual knowledge to skill and behavior is less clear. Based on cognitive development theories, a new concept, “awareness as connection”, was devised, and a questionnaire addressing the connection between food, nutrition, diabetes, and behavior was developed to assess the awareness of adolescents. Methods: A lesson designed to connect food, nutrition, and local lifestyle was piloted with a group of 146 students aged 13–17 years in northern Taiwan. Results: The pre- and posttest results found that students improved regarding their awareness of the association between food, nutrition, diabetes, and lifestyles (*p* < 0.01). The exploratory factor analysis results revealed one factor for the pre-test but two factors for the posttest, indicating growth in the students’ knowledge structure. Most of the students demonstrated significant progress in the posttest, especially on the knowledge application subscale. The results reveal that students in lower grades started low and ended low but achieved greater gains than high schoolers. High-school students had better nutritional knowledge, but junior-high-school students made substantial progress on the application of this knowledge, indicating an improvement for cognitive complexity. Students with overweight started out low and achieved fewer and uneven gains than students without overweight but scored high on particular items. Gender did not have significant impact on the overall learning, but girls showed more gains in factual knowledge. Conclusions: These results suggest that awareness can be a mid-step to help link knowledge to behavior, and for some, this step may take more time.

## 1. Introduction

### 1.1. Missing Links Between Knowledge and Behavior

The population of young people with metabolic illness is on the rise in many countries, especially regarding the incidence of type 2 diabetes [1,2]. In Lawrence et al.’s study [1], the prevalence of type 2 diabetes among those aged 10 to 19 years almost doubled, increasing from 0.34 to 0.67 per 1000 youths from 2001 to 2017. Factors such as the modern sedentary lifestyle, obesity, and family history contribute to this trend [3]. Educating young people about food and nutrition has been regarded as important, but the way to develop understanding and encourage the practice of a heathier lifestyle is still unclear.

Knowledge of food, nutrition, and healthy lifestyles has been found to be a crucial factor in healthier food consumption and a lower rate of metabolic illness [4,5], but declarative knowledge alone may not be sufficient to change behavior [6]. Many educational programs have been planned to change behavior right after an educational intervention. The theory of planned behavior and social cognitive models of knowledge, attitude, and behavior has been widely used to guide the design of programs and tools to assess the effectiveness of food and nutrition interventions [7]. However, some researchers have suggested that the link between knowledge and behavior is not exactly apparent. Ronto et al. [8] conducted interviews with adolescents aged 12–17 years with quantitative and qualitative questioning. Although the adolescents stated that food and nutrition knowledge is important for them to eat well, the majority did not convert their knowledge to practice.

The lack of application from knowledge to behavior can be found in large samples. Bellisle et al. [9] surveyed 1000 French children aged 9–11 years and found that the children could list “healthy foods” competently, but their preferred food was often rich in sugar and/or fat (including fried potatoes, ice cream, nut spread, chocolate, and cake). Another study on 638 college students regarding the consumption frequency of 24 food groups found that declarative knowledge of nutrition does not correlate with behavior. This suggests that education programs should also promote favorable attitudes towards food and nutrition [6].

Interventions to change behavior can be more challenging in some cases, such as for children with overweight [10]. In a seven-month school food and nutrition program developed using social cognitive theory, 172 high school girls with overweight and obesity attended the program’s workshops alongside their teachers and parents. The results did not reveal significant changes in the BMI or WCs of the participants, but their dietary habits and psychological factors (self-efficacy, social support, and intention) improved significantly in the intervention group [11]. It is possible that in between knowledge and behavior, there are other issues involved, such as attitude and skill.

### 1.2. Proposing Awareness as a Step to Connect Knowledge to Attitude and Behavior

In between the conversion of knowledge to behavior, there could be many more steps or connections than previously envisioned. At least three areas of knowledge expansion or connections are still needed:

1. Knowledge connection. Researchers found that even after school education, adolescents demonstrate only spotty knowledge about food and nutrition [12,13]. For example, adolescents were able to correctly categorize fruit and cola as “healthy” or “unhealthy” food but were unable to categorize processed foods or answer correct portion sizes [14]. Moreover, adolescents have limited cognitive development and difficulty connecting food to health and to nutrition [15]. Even older students proficient with nutrition knowledge did not apply their knowledge in their daily lives [12];
2. Skill connection. Studies have attributed the inability of adolescents and college students to implement their nutritional knowledge into their daily lives to a lack of procedural knowledge, such as meal preparation [8,16]. The procedural areas can broadly include functional skills such as preparing food, finding locally available food, making decisions based on the food choices available, cooking, and budgeting [17,18]. This area is often connected to and situated in ones’ own local environment or sociocultural practices [19];
3. Attitude connection. Even students that have nutrition knowledge may lack the willpower or motivation to implement it [20,21]. Heathy eating attitudes and behavior can be promoted through a positive attitude to or connection with healthy food, such as through parents’ encouragement, experience gardening, or cooking capabilities [22,23,24]. Moreover, concerns about appearance for girls or participation in sports for boys can also be driving forces for healthy eating [25]. Students with diabetic family members may also have heightened awareness and score high on food and nutrition knowledge and food label reading skills [26,27].

In summary, there could be more steps in the process leading to behavioral change. However, although behavioral change is a long-term goal, attitudinal change can be placed earlier in the process. Moreover, because a change in attitude can result from the accumulation of knowledge [28], the focus could be first placed on the connection between knowledge and other areas.

### 1.3. Constructing the Awareness Lesson and Scale for Adolescents

This study proposes the concept of “awareness as connection” as an intermediate step or process, utilizing an idea proposed by Lane and Schwartz [29], who based their research on Piaget and Werner, that knowledge should be made to connect to learners’ physiological and emotional reactions to increase awareness. Food and nutrition knowledge needs to be connected to student behavior and lifestyle for students to make the link between the knowledge and their own condition. Using diabetes prevention as a theme to focus on this association, more explicit links were implemented in lessons and assessments to create an educational project for adolescents.

**Figure 1:**
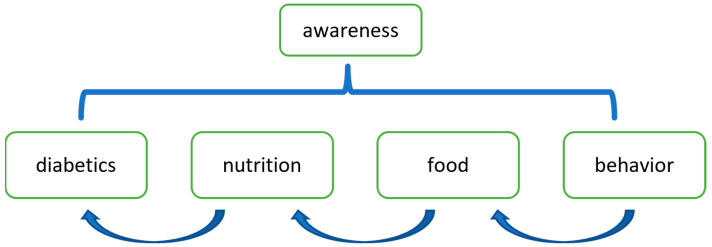
A schematic representation of the program “awareness as connection”.

The program “awareness as connection” was developed based on the following theoretical underpinnings (Figure 1). The goal is to raise awareness by enhancing the connection between knowledge and behavior in their local situations to increase positive attitudes.

#### 1.3.1. Making Connections Encourages Complex Thinking and Autonomy in Adolescents

Understanding of food and nutrition emerges early in development. Preschool-aged children conceptualize food in terms of its edibility, nutritional value, and digestive processes, informed by their direct experiences with eating [30]. When children mature, the complexity of their thinking increases. Younger children tend to hold practical and concrete perceptions of food and nutrition [15], whereas adolescents develop more abstract reasoning, forming complex associations with various dimensions of food and nutrition [31]. Older school-aged children and adolescents, for example, often express concerns about dietary choices, obstacles to maintaining a healthy lifestyle, and physical appearance [30]. This project builds on the natural tendency of adolescents seeking to expand their horizons.

Moreover, adolescents may cultivate independent perspectives on food choices that diverge from familial practices established in childhood [32]. In situations where parents exercise greater control, adolescent children could have more rebellious reactions [33,34]. Ultimately, adolescents exercise autonomy in their dietary decisions. Research indicates that adolescents’ self-determined motivation significantly influences their consumption of healthier foods and beverages, independent of external motivational factors [23]. Reflecting on one’s eating habits and lifestyle facilitates one’s own decision making.

#### 1.3.2. Building Links Between Knowledge and Behavior to Foster Awareness

Research has indicated that declarative knowledge alone does not predict behavior well [6,9]. Procedural or functional knowledge as well as critical analysis capability should be part of food system knowledge [17,35]. Therefore, people may not only need to know the “what” but also the “how”, which can be seen as the application of declarative knowledge to their own situations, such as food markets or dinners or the manner of eating [10].

Currently, research on food and nutrition literacy includes many important topics, such as the nutritional value of food items, food labeling, and sanitary requirements [8], but these topics are rarely seen as connected. Making these links could create a comprehensive web from knowledge to application. If these linkages are not articulated by their family or school, students’ understanding of these elements can be spotty [12,13].

#### 1.3.3. Using Disease as a Theme to Focus Awareness

Disease prevention programs for raising awareness can be modeled because their goal is focused. A program targeting a high-risk population to reduce the risk of diabetes may cover topics such as nutrition knowledge, the impact of obesity, and the treatment of diabetes [36]. Adolescents can also benefit from learning that sitting too long may cause heart problems, eating irregularly may cause diabetes, and kidney dialysis is a complication for diabetes [37,38].

This project makes a deliberate connection between knowledge, the life of adolescents, and the likelihood of developing diabetes. In the lesson design, connections between food, nutrition, lifestyle, and diabetes are explicitly drawn. The lesson explains how glucose levels in the body connect to food categories, nutrition, eating habits, exercise, body weight, stress, and sleeping patterns. At the end of the lesson, diabetes impacts on one’s health, finances, employment, and life expectancy are discussed [39].

## 2. Materials and Methods

### 2.1. Method and Setting

To test the validity of the questionnaire, a quasi-experimental design with pre- and posttests was implemented [40]. A one-day lesson of six hours was delivered to students, and a questionnaire was filled out before and after the lesson.

Activities to increase the students’ awareness of the food and nutrition were built into the lesson. During the discussion of food and nutrition, students were asked about what they eat for breakfast. During the part of the lesson where the danger of being overweight was emphasized, students were provided with measuring tapes to measure their waists and calculate their BMI.

### 2.2. Participants

A total of 146 students aged 13–17 years and from three local schools in northern Taiwan participated in this study. There were a few more junior-high-school students (grades 7–9) (n = 88) than senior-high-school students (grades 10–11) (n = 58), and a few more boys (n = 79) than girls (n = 67). Based on those who provided daily energy needs (n = 141) [41], the minimum was 1440 kcal, the maximum was 3516 kcal, and the average was 1888 kcal.

### 2.3. Instrument

A 14-item Awareness Questionnaire was developed to address the connections between food, nutrition, diabetes, and behavior based on the lessons taught (Table 1). Attention was given to question design that makes specific connections between knowledge and local food choices and common eating practices. A panel of school teachers and nutritionists reviewed and made suggestions to the questionnaire items.

Additional information about the participants was collected in the questionnaire, including grade level, gender, and daily nutritional needs. The daily nutritional need was calculated based on the age, height, weight, and activity level. Answering these additional questions was optional for students.

The items were in true–false format. The students could select “don’t know” as a third choice. Only items answered correctly were marked as correct. The correction rate for each item was calculated across all students by taking the number of students who answered the item correctly over the total number of students.

### 2.4. Analysis

Statistical package IBM SPSS version 22 was used for data analysis. Exploratory factor analyses (EFAs) were performed for both the pre-test and posttest to explore the latent structure of the awareness scale. Principal axis factoring and direct oblimin rotation were chosen for the EFAs because of the potential correlation of the factors.

For data normality, skewness and kurtosis were examined. Although the correction rates for the pre-test were roughly normal, with skewness and kurtosis values between −1 and +1 (skewness = −0.76, kurtosis = 0.32) [40], the posttest showed a more negative skew (skewness = −1.06, kurtosis = 0.55) due to a concentration of higher scores.

For the students’ gains from the treatment, a paired *t*-test was used to examine the differences before and after the treatment. Factors such as age, gender, and daily energy needs were also analyzed for the applicability of the questionnaire.

## 3. Results

### 3.1. Exploratory Factor Analysis (EFA) of the Awareness Questionnaire

The EFA for the pre-test showed that one factor was sufficient to represent the structure. The total variance explained by one factor is 16%, which is not very high. On the other hand, the KMO measure of sampling adequacy was greater than 0.6 (KMO = 0.671), and Bartlett’s test of sphericity was significant (*p* < 0.001). Moreover, a low root mean square residual (RMSR = 0.07) also indicates a low average residual between the observed and reproduced correlations and a proper fit of the structure to the data. Four items (#6, #10, #12, and #13) had low factor loadings (<0.30), which may be considered as insufficient fitting.

### 3.2. Students’ Performance on the Awareness Questionnaire

Based on the one-factor structure, students’ performances in terms of the correction rate are listed in Table 2. Using the paired *t*-test, students’ scores before and after the treatment were calculated. Overall, students achieved great gains in awareness from the lesson (*t* = 4.729, *p* < 0.001). Students of different age groups (junior high and senior high school) all made progress, and both boys and girls improved in their awareness of food and nutrition.

Overall, high-school students had a good level of knowledge at the beginning and end of the class. In comparison, junior-high-school students started at a low knowledge level (mean difference = −0.10, *t* = 2.95, *p* < 0.01) and ended low (mean difference = −0.07, *t* = 2.22, *p* = 0.03) but demonstrated good gains from the program (*t* = 3.93, *p* < 0.001). The mean difference between boys and girls was minimal both in the pre-test (mean difference = 0.01, *t* = 0.44, *p* = 0.66) and posttest (mean difference = 0.05, *t* = 1.59, *p* = 0.12).

Additional attention was given to the awareness level of students with high daily nutritional energy needs. Since participants were about the same age, those whose daily nutritional energy needs exceeded 2100 kcal were considered overweight. In the pre-test, those who were overweight had the lowest awareness score among various background factors. Their posttest scores improved, but the gain was the smallest and on the verge of being significant. Those who did not provide nutritional energy need information were not included in the analysis.

### 3.3. Posttest EFA

Another exploratory factor analysis was performed for the posttest. Two subscales emerged as a result (Table 3). Both subscales explained 28% of the variance, which is a great improvement from the pre-test EFA and is considered acceptable for social science studies [40]. This structure also had a low root mean square residual (RMSR = 0.06), meaning the two-scale structure is also a good fit for the data. Most of the items have good factor loadings, except two items (#9 and #12) with smaller values.

The results suggest that the participants may view the items in the Awareness Questionnaire differently before and after the lesson. Before the lesson, their ideas about these elements were more scattered or unformed, but after the lessons, they began to see these items in a more structured way.

Upon examining the two subscales, items related to food and nutrition fall into subscale 1 (7 items). Items that connect more broadly to nutrition, diabetes, and behavior belong to subscale 2 (7 items), and items in subscale 2 can also be seen as applications of food and nutrition knowledge to behavioral decisions or diabetic prevention measures.

Further examination of students’ performance on the two subscales on the Awareness Questionnaire, based on the posttest EFA analysis, proved that students’ existing knowledge in food and nutrition was adequate before the lesson (Table 4). Students’ performance on subscale 1 saw improvement but did not make significant progress. Most of the changes occurred in subscale 2, which concerns the application of food and nutritional knowledge to behavior or disease (overall gain = 0.15, *t* = 6.48, *p* < 0.001).

Students of various backgrounds and across age, gender, and weight situations exhibited the same pattern for subscale 1 and subscale 2. After the lesson, they did not make much progress on subscale 1 but made significant progress on subscale 2. Specifically, the senior-high-school students’ score was very high in subscale 1, so not much progress could be made there, but they exhibited much progress on subscale 2 (*p* < 0.01). Again, the junior-high-school students started lower and ended lower on both subscales compared to the high schoolers, but they made the most progress in subscale 2 (*t* = 6.03, *p* < 0.001).

Boys and girls exhibited different patterns of learning on subscales 1 and 2. Boys’ improvement on subscale 1 was minimal, with the same mean scores for the pre- and posttest, but their gains on subscale 2 were substantial (*t* = 3.88, *p* < 0.001). Girls, conversely, made the most progress in subscale 1 (*t* = 2.20, *p* = 0.03) and also performed well on subscale 2 (*t* = 5.31, *p* < 0.001).

Students who were overweight made progress on both subscales 1 and 2, but their progress for subscale 2 was relatively small and variable. Perhaps partially due to the small sample size, their progress did not achieve statistical significance [40].

**Figure 2:**
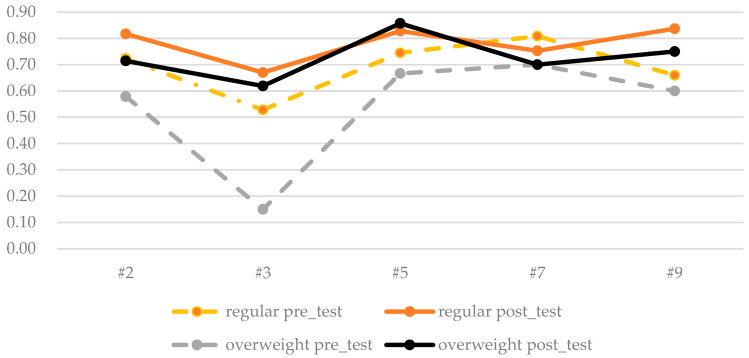
Graphic plot of the pre- and posttest items for regular and overweight students.

To examine the situation of the students with overweight, some items that connected the concept for food, nutrition, behavior, and diabetes were taken out for additional analysis. Graphs were plotted for the mean correction rate for the pre- and posttests for regular and overweight subjects. The items taken for comparisons are listed in Table 5, and the pre-test and posttest means are depicted in Figure 2.

Before the class, students with overweight had a general understanding about the health value of unprocessed food, but they understood less about detailed applications to daily lives or illness, such as in making choices between fruit and fruit juice or that snacking between meals can cause insulin fluctuation. After the class, these students demonstrated good progress. In some items, such as #5, they even excelled, but they performed the same for other items, such as #7: “Eating dessert before or after a meal has the same effect on the body’s glucose levels”.

## 4. Discussion

### 4.1. Measuring Awareness-as-Connection Is a Viable Goal for a Program

Past research has found a discrepancy between food knowledge and food skills [21,42] and proposed that there is a need for both in food and nutrition literacy [17]. This disconnection of food and nutrition knowledge could indicate a need to apply knowledge to behavior in the context of the local environment and cultural traditions [19]. Moreover, linking this to future illness expands the implications of food and nutrition knowledge and further motivates adolescents to adopt a healthy lifestyle [36]. Viewing food and nutrition literacy as cognitive expansion [21,42], this study aimed to find a step between knowledge and behavior.

Based on cognitive development theory and cognitive complexity [29,43], this study proposes the awareness-as-connection framework that makes a deliberate effort to connect knowledge of food and nutrition to local behavioral choices and illness implications. The results of the exploratory factor analysis show that the 14-item Awareness Questionnaire is adequate as a one-factor structure, especially before the students completed the lesson. After the lesson, the students demonstrated improvement as measured using the Awareness Questionnaire and seen across various student groups. The wide range of improvement also shows the validity of the questionnaire.

Intervention programs regarding food and nutrition education and diabetic prevention largely focus on changing student behavior [7,44], but their effect may not be immediately apparent [45], especially for those who have overweight conditions [11]. This study proposes that changing the awareness of the relationship between food, nutrition, behavior, and illness can also be a viable goal. These connections of knowledge may change behavior in the long run.

### 4.2. Awareness Enhances Learning About Knowledge and Application

This study also found that students’ knowledge structures changed after the lesson on the awareness of food and nutrition and its application to daily lives and illness. Maintaining the overall structure as adequate (RMSR = 0.06), the posttest EFA revealed two subfactors after the lesson implementation, and the percentage of variance explained improved from 16% to 28%. The results suggest that after the lesson, students may have been able to better differentiate concepts and form distinct constructs during the posttest [46].

Two subscales were identified. For the first subscale, regarding food and nutrition knowledge, the students seemed to have good existing knowledge from previous education as well as maturity [31,47], especially the high-school students. Conversely, students’ progress on the second subscale was much more substantial. Students across groups made significant progress in this category. The application of nutrition knowledge to behavioral decisions and diabetes assessment was the main gain in the awareness scale. As Ronto et al. pointed out, students may not be able to apply their knowledge in practice due to lack of confidence [8]. The lesson designed with the awareness framework may provide the confidence needed for application.

The result of this research echoes recent efforts to delineate knowledge and competences regarding food and nutrition with the growth of cognitive complexity [10,17]: As children grow and become adolescents, their interpretation of food may move from sensational or relational to more complicated functional knowledge and skills, and then, with wider exposure, analytical views or critical judgment may develop [10,17]. Through this process, adolescents can gain greater autonomy in their food choices and increased agency in responding to environmental influences [10,17].

School curriculum, education programs, and awareness programs offer helpful content, but these interventions seem to be concentrated mainly in one area [48]. Making deliberate efforts to associate these concepts with locally situated behavior and judgments of potential illness has the potential to enhance students’ cognitive complexity in food and nutrition.

### 4.3. The Awareness Gains of Different Groups

Assessed using the Awareness Questionnaire, all but one group of students of various backgrounds made significant progress after the program. A closer look at the results of the different groups further reveals the usefulness of the questionnaire.

#### 4.3.1. Age

Although some survey studies have found senior-high-school students to have lower food and nutritional knowledge than junior-high students [49], in general, education matters, and older students possess higher education and more knowledge in food and nutrition [50,51]. As evidenced by this study, high-school students demonstrated greater nutrition knowledge than younger adolescents. However, their association of such knowledge with daily situations and potential illness still has room for improvement [15,52]. In this study, this knowledge application area is where they improved the most.

The finding that junior-high-school participants improved the most with the program is very encouraging. In one study, children’s nutrition knowledge level in the lowest quartile increased substantially but not in a higher quartile [28]. Similarly, in this study, the junior-high-school students’ knowledge was good but not great, but they made the largest gains overall, especially in the application area of subscale 2. Given properly designed lessons and material, it is possible for younger students to grow in cognitive complexity in food and nutrition.

#### 4.3.2. Gender

Previous studies have found that girls have higher nutritional knowledge, make better food consumption choices, and care more about appearances [25,53]. In this study, for overall awareness, both girls and boys made significant progress on their awareness after the program. However, in looking closer into the two subscale scores on the posttest, girls made higher gains on subscale 1, where boys made no gains at all. This result may reflect that male students may lack motivation for healthy dieting [20,21] but are more interested in topics that involve sports or interactive activities [8].

Both girls and boys, in addition to nutritional knowledge, need skills and procedure knowledge [15]. In this study, both made significant progress on subscale 2 in knowledge application. Girls scored slightly lower than the boys at the start, but they did as well as the boys at the end, resulting in no gender differences in the overall scores. Ashoori et al. also found some gender differences on some parts of the food and nutrition scale, but no difference was observed in the overall gains of the boys and girls [26,27].

#### 4.3.3. Overweight

Students with overweight represented a small number of participants, but their results are noteworthy. Although the mean scores on the Awareness Questionnaire (Table 2) show that students with overweight made progress overall, they did not perform as well as other students. As indicated by previous research, changing the thinking and behavior of high-school students with overweight takes time and effort, even with the involvement of school and parents [11].

Further examination of the two subscales from the posttest EFA analysis (Table 3) suggests that students with overweight already possessed good knowledge on the first subscale. However, they made limited progress on subscale 2, which is about the application of knowledge. The lack of significance for subscale 2 may be due to the small sample size and high sampling variability, but the low correction rate at the start suggests a low level of knowledge for these students. Moreover, the detailed item analysis in Figure 2 shows that they are particular and selective in the things they learn well. Past research has found that adolescents with low dietary knowledge levels may be associated with overweight and obesity [4,5], which is in line with the current findings. The results of this study, however, suggest that in comparison to food and nutrition knowledge, it is the application of such knowledge to behavior and illness that should be stressed. To assist students with overweight, providing procedure knowledge tailored to their local settings and focused on their particular needs could be beneficial [54].

### 4.4. Research Limitations

This study proposed a framework to raise adolescents’ awareness of food and nutrition, designed a corresponding lesson and questionnaire, and conducted workshops to evaluate the outcomes and validate the instrument. The result was encouraging. However, several limitations should be acknowledged. First, the sample size of 146 students, while meeting the minimum 10:1 participant-to-item ratio for exploratory factor analysis (EFA) with a 14-item questionnaire [40], may limit statistical power for detecting small effect sizes in *t*-tests and the stability of EFA factor structures. Second, this study was conducted in only three schools located in northern Taiwan, which may limit the generalizability of the findings. Third, very few students with overweight were included in the sample. Although this study was not specifically aimed at overweight populations or disease prevention—conducted in the context of a high-school camp or workshop—this limitation considerably affects the validity and applicability of the results. Future research with larger, more geographically diverse samples is recommended to enhance reliability and applicability of the instrument.

## 5. Conclusions

Using cognitive development theory as a foundation, this study explored the use of an Awareness Questionnaire for adolescents that addresses the connection between food, nutrition, behavior, and diabetes. The awareness-as-connection framework brings together knowledge and skill and places them on the same continuum as knowledge and application. The Awareness Questionnaire intentionally integrates food and nutrition knowledge with the food items and behavioral decisions usually encountered by local students. The exploratory factor analysis of the pre-test revealed one factor, and the posttest revealed two factors. This result implies that the students’ knowledge about food and nutrition was less differentiated before the lesson and that they formed a more structured view about the same questions after the lesson. The results of the analysis of the means of different groups, including age, gender, and weight, are in line with past research and further validate the Awareness Questionnaire. These results suggest that the Awareness Questionnaire is a viable alternative with which to assess the results of education programs about food and nutrition and delineates a middle step in behavioral change. Nevertheless, the connection of knowledge to behavior and illness is an area that needs additional exploration in order to improve students’ learning regarding food and nutrition.

## Figures and Tables

**Table 1:** The associations between elements for the items ^1^.

#	Item	Food	Nutrition	Diabetes	Behavior
1	Milk tea and Chinese omelet are a nutritionally balanced breakfast choice.	V	V		
2	Refined starches are more nutritious and healthier than unprocessed whole grains and cereals.	V	V		
3	Eating vegetables first and then rice results in a smaller rise in blood sugar levels.	V		V	V
4	Replacing regular meals with fruit can help people eat less while staying healthy.	V	V		V
5	Eating fruit and drinking fruit juice results in the same speed of glucose conversion in the body.	V	V	V	
6	Sugar-free drinks from convenience stores will not make you gain weight or cause any health burdens.	V		V	V
7	Eating dessert before or after a meal has the same effect on the body’s glucose levels.	V		V	V
8	Chewing food slowly and thoroughly helps the body achieve a steady blood sugar level.			V	V
9	Snacking between meals causes insulin to be frequently secreted in large amounts.			V	V
10	If the body does not feel unwell, it indicates that the blood sugar level is very stable.		V	V	V
11	If fasting blood sugar exceeds 120 mg/dL, it may indicate the presence of diabetes.		V	V	
12	The daily nutritional needs of teenagers are related to their activity levels.		V		V
13	Exercise helps the body burn energy and helps reduce blood sugar fluctuations.		V	V	V
14	You can tell if blood sugar is gradually becoming uncontrolled by looking at waist circumference. If the waist circumference exceeds 90 cm, there is a risk of diabetes.		V	V	V

^1^ The conceptual categories which the items belong to are marked with a “V”.

**Table 2:** Students’ performance on the Awareness Questionnaire (14 items) ^1^.

	Pre-Test		Posttest			
Subjects	Mean	SD	Mean	SD	*t*	*p*
All (n = 146)	0.70	0.20	0.78	0.19	4.729	<0.001
Junior H. ^2^ (n = 88)	0.66	0.20	0.76	0.20	3.934	<0.001
Senior H. (n = 58)	0.76	0.17	0.83	0.16	2.617	0.011
Boys (n = 79)	0.69	0.21	0.76	0.21	2.415	0.018
Girls (n = 67)	0.71	0.18	0.81	0.17	5.056	<0.001
Overweight (n = 21)	0.61	0.21	0.73	0.23	2.038	0.055
Regular weight (n = 94)	0.71	0.20	0.79	0.20	3.570	0.001

^1^ The performance is the correction rate of all items averaged across the student groups. ^2^ Junior H. = Junior High schoolers; Senior H. = Senior High schoolers.

**Table 3:** EFA of posttest.

	Factor Loading ^1^		Subscale	
Item #	subscale 1	subscale 2	subscale 1	subscale 2
1	0.405	0.167	1	
2	0.550	0.171	1	
3		0.247		2
4	0.582		1	
5	0.715	0.265	1	
6	0.605	0.102	1	
7	0.616	0.156	1	
8		0.320		2
9	0.164	0.291		2
10	0.551	0.255	1	
11	0.165	0.563		2
12	0.162	0.371		2
13	0.294	0.463		2
14	0.211	0.683		2

^1^ Factor loadings less than 0.10 are not listed.

**Table 4:** Students’ performance on the posttest’s two scales.

	Subscale 1								Subscale 2			
	Pre-Test		Posttest				Pre-Test		Posttest			
	M	SD	M	SD	*t*	*p*	M	SD	M	SD	*t*	*p*
All (n = 145) ^1^	0.80	0.21	0.82	0.25	1.18	0.24	0.60	0.25	0.75	0.24	6.48	<0.001
Junior H. ^2^ (n = 87)	0.78	0.24	0.80	0.27	0.99	0.33	0.57	0.22	0.73	0.24	6.03	<0.001
Senior H. (n = 58)	0.85	0.15	0.87	0.20	0.65	0.52	0.67	0.26	0.78	0.22	2.90	0.005
Boys (n = 78)	0.79	0.22	0.79	0.27	0.12	0.90	0.62	0.25	0.75	0.24	3.88	<0.001
Girls (n = 67)	0.82	0.20	0.87	0.21	2.20	0.03	0.60	0.24	0.76	0.22	5.31	<0.001
Overweight (n = 20)	0.73	0.17	0.77	0.29	0.63	0.54	0.53	0.27	0.67	0.27	2.00	0.061
Regular (n = 94)	0.81	0.22	0.83	0.24	0.81	0.42	0.63	0.24	0.81	0.23	5.13	<0.001

^1^ One participant had more than five missing data points in the posttest and was excluded from the EFA. ^2^ Junior H. = Junior High schoolers; Senior H. = Senior High schoolers.

**Table 5:** Selected items for mean comparisons for connected concepts.

Item #	Question	Subscale
2	Refined starches are more nutritious and healthier than unprocessed whole grains and cereals.	1
3	Eating vegetables first and then rice results in a smaller rise in blood sugar levels.	2
5	Eating fruit and drinking fruit juice result in the same speed of glucose conversion in the body.	1
7	Eating dessert before or after a meal has the same effect on the body’s glucose levels.	1
9	Snacking between meals causes insulin to be frequently secreted in large amounts.	2

## Data Availability

The data presented in this study are available on request from the corresponding author due to privacy reasons.
